# The S-Nitrosylation Status of PCNA Localized in Cytosol Impacts the Apoptotic Pathway in a Parkinson’s Disease Paradigm

**DOI:** 10.1371/journal.pone.0117546

**Published:** 2015-02-12

**Authors:** Liang Yin, Yingying Xie, Songyue Yin, Xiaolei Lv, Jia Zhang, Zezong Gu, Haidan Sun, Siqi Liu

**Affiliations:** 1 Key Laboratory of Genome Sciences and Information, Beijing Institute of Genomics, Chinese Academy of Sciences, Beijing, China; 2 University of Chinese Academy of Sciences, Beijing, China; 3 Beijing Protein Innovation, Beijing, China; 4 Department of Pathology and Anatomical Sciences, University of Missouri School of Medicine, Columbia, Missouri, United States of America; Hokkaido University, JAPAN

## Abstract

It is generally accepted that nitric oxide (NO) or its derivatives, reactive nitrogen species (RNS), are involved in the development of Parkinson’s disease (PD). Recently, emerging evidence in the study of PD has indicated that protein S-nitrosylation triggers the signaling changes in neurons. In this study, SH-SY5Y cells treated with rotenone were used as a model of neuronal death in PD. The treated cells underwent significant apoptosis, which was accompanied by an increase in intracellular NO in a rotenone dose-dependent manner. The CyDye switch approach was employed to screen for changes in S-nitrosylated (SNO) proteins in response to the rotenone treatment. Seven proteins with increased S-nitrosylation were identified in the treated SH-SY5Y cells, which included proliferating cell nuclear antigen (PCNA). Although PCNA is generally located in the nucleus and participates in DNA replication and repair, significant PCNA was identified in the SH-SY5Y cytosol. Using immunoprecipitation and pull-down approaches, PCNA was found to interact with caspase-9; using mass spectrometry, the two cysteine residues PCNA-Cys81 and -Cys162 were identified as candidate S-nitrosylated residues. In addition, the evidence obtained from in vitro and the cell model studies indicated that the S-nitrosylation of PCNA-Cys81 affected the interaction between PCNA and caspase-9. Furthermore, the interaction of PCNA and caspase-9 partially blocked caspase-9 activation, indicating that the S-nitrosylation of cytosolic PCNA may be a mediator of the apoptotic pathway.

## Introduction

Parkinson’s disease (PD), the second most common neurodegenerative disorder, is characterized by a massive and specific loss of dopaminergic neurons in the substantia nigra pars compacta (SNpc) [1, 2]. The exact causes of this neuronal loss have not yet been fully elucidated, but a large body of evidence has revealed the major contribution of apoptosis [3]. Programmed cell death is a normal process, whereas apoptosis can trigger several pathological changes, including neurodegenerative disorders. Interestingly, apoptotic neurons have been identified in the SNpc of PD patients via an in situ end labeling method [4], and the activation of caspases has been detected in the nigral neurons of a PD mice model [5]. Many mechanisms are likely involved in neuronal apoptosis, including oxidative stress, mitochondrial dysfunction, energy imbalance, inflammation, defects in familial genes, and dysfunction of the ubiquitin-proteasome system (UPS). It is difficult to attribute neuronal apoptosis to a single causal factor as hypothesized by Sulzer, the neurodegeneration in PD likely results from “multiple hits” [6]. Therefore, an intensive investigation of PD focusing on the detailed events related to the pathological process is urgently required.

Nitric oxide (NO) is a free radical in a highly diffusible gaseous state and is regarded as an important regulator for numerous biological processes, such as vasodilation [7], neurotransmission [8], and inflammatory responses [9]. Excessive production of NO and NO-derivative reactive nitrogen species (RNS) has been implicated in neuron damage, particularly neurodegeneration in PD [10–12]. Increased expression of iNOS has been identified in PD animal models induced by 6-OHDA and LPS or rotenone, leading to increased NO levels and lipid peroxidation products [13–15]. The overexpression of nNOS and the generation of peroxynitrite (ONOO^-^) have been observed in PD patients [16]. It is generally accepted that RNS are highly active and able to react with many macromolecules. S-Nitrosylation, which is a reversible covalent addition of a NO group to a cysteine residue’s sulfhydryl that forms S-nitrosothiols, is a typical protein modification induced by RNS [17]. Several investigators have focused on the correlation between S-nitrosylation and PD. For example, the cysteine residue at 644 in dynamin-related protein 1 (Drp1), a member of the Dynamin family of large GTPases, is likely to be S-nitrosylated (SNO). The S-nitrosylation of Drp1 promotes its multimerization, leading to mitochondrial fission and neuronal damage [18]. In addition, the cysteine residues at 51 and 172 in peroxiredoxin 2 (Prx2), an antioxidant protein, are S-nitrosylated. In neuronal cells, SNO-Prx2 becomes inactivated, sensitizing the cells to oxidative stress-dependent cell death [19]. S-Nitrosylation has a direct role in regulating protein functions and affects protein interactions through structural alterations. The S-nitrosylation of GAPDH enables its binding and stabilization of the E3 ubiquitin ligase Siah1, thus facilitating nuclear protein degradation and stimulating apoptotic cell death [20]. In addition, the S-nitrosylation of the BIR domain in X-linked inhibitor of apoptosis protein (XIAP) decreases its binding to caspase-3, which is accompanied by the loss of the anti-apoptotic ability of XIAP under nitrosative stress [21]. However, no general mechanism for the functional changes of SNO proteins in PD has been identified to date.

SH-SY5Y, a cell line derived from a female neuroblastoma patient, is generally used as in vitro model of neuronal function and differentiation. Because this cell line has an adrenergic phenotype with dopaminergic markers, it has been extensively accepted in the study of PD [22]. Rotenone, a pesticide that functions as a selective inhibitor of complex I, is well recognized to induce apoptosis via the caspase-dependent apoptotic pathway in neuronal cells, such as SH-SY5Y. One of the proposed mechanisms of rotenone neurotoxicity is oxidative stress via the production of reactive oxygen species (ROS), such as superoxide anion, hydroxyl radicals, and hydrogen peroxide, which initiates a ROS-related series of reactions, such as lipid peroxidation, protein and DNA oxidation, and variations in the ratio of reduced to oxidized glutathione (GSH) [23]. **C**hronic low-dose rotenone treatment has been observed to induce the excessive accumulation of ROS, inclusion body formation and apoptosis in dopaminergic neurons of animal and human origin; furthermore, SH-SY5Y cells treated with rotenone exhibit a 2-fold increase in intracellular ROS compared with untreated controls [24]. Moreover, recent studies have reported that the rotenone treatment of SH-SY5Y cells augments the generation of RNS and protein modifications caused by RNS, whereas the inhibition of nitric oxide synthase attenuates rotenone-induced apoptosis [15, 19, 25]. Generally, it is believed that rotenone can cause neuronal depolarization, resulting in activation of N-methyl-D-aspartate (NMDA) glutamate receptor, a voltage dependent Ca^2+^ channel. Since neuronal nitric oxide synthase (nNOS) is a Ca^2+^ dependent NOS, increased Ca^2+^ due to rotenone treatment thus enables activation of nNOS, leading an increase of NO production. In addition, NO has been shown to S-nitrosylate caspases-3 [26], -8 [27], and-9 [28] at the active site cysteine, thus inhibiting enzymatic activity and affording neuroprotection. How the S-nitrosylation of caspases influences the apoptotic process remains unclear. Thioredoxin2 (TRX2) has been regarded as a transnitrosylation mediator that selectively denitrosylates the S-nitrosothiols of caspase-3 [29], whereas another study proposed that the neuroprotective activity could be abrogated during nitrosative stress via transnitrosylation between XIAP and caspases [30]. According to that model, transnitrosylation from SNO-caspases to XIAP acts as an apoptotic switch. The ubiquitination and target degradation functions of XIAP are regulated by its S-nitrosylation status. Therefore, RNS have dual functions, namely, the direct inhibition of caspase activity by S-nitrosylation and the attenuation of caspase degradation by XIAP via SNO-XIAP formation. The mechanism of RNS involvement in apoptosis of rotenone-treated SH-SY5Y cells is unclear.

In this research, we used rotenone-treated SH-SY5Y cells as a typical PD model to investigate the mechanism between SNO proteins and neuronal apoptosis. The canonical approach of SNO proteins detection is biotin switch technique, from which many proteomic strategies were derived for S-nitrosoproteome analysis [31]. Herein, after carefully estimating the nitrosative stress and apoptosis in SH-SY5Y cells, we utilized fluorescence-tagged CyDye thiol reactive agents to label S-nitrosothiols on SNO proteins, which was combined with two-dimensional difference gel electrophoresis (2D-DIGE) to separate the SNO proteins. Approximate two hundred fluorescent spots were detected via image analysis; in these spots, 7 proteins were identified as having increased S-nitrosylation in response to rotenone treatment. We focused on the physiological role of the S-nitrosylation of PCNA because this protein is closely related to the cell cycle and the regulation of apoptosis. Its S-nitrosylation was coincidentally up-regulated in another study of PD neuronal model [32]. Interestingly, we observed that PCNA in SH-SY5Y cells was predominately localized in the cytosol and was able to interact with caspase-9. Our data further demonstrate that the up-regulated S-nitrosylation of PCNA decreases the interaction of PCNA and caspase-9, leading to an increase of caspase-9 cleavage, so called caspase-9 activation. The active form of caspase-9 is expected to activate the apoptotic pathway in dopaminergic neuron in PD. This is a reasonable deduction derived from our observation how PCNA S-nitrosylation performs a significant impact to the functions of SH-SY5Y cells, leading to a regulatory mechanism of cell death in PD.

## Materials and Methods

### Reagents

Rotenone, N^G^-Methyl-L-arginine acetate salt (L-NMMA), S-Methyl methanethiosulfonate (MMTS), Sodium L-ascorbate, Neocuproine and DMF were purchased from Sigma-Aldrich. 4-Amino-5-Methylamino-2′,7′-Difluorofluorescein Diacetate (DAF-FM DA) were obtained from Molecular Probes. Biotin-HPDP and NeutrAvidin agarose were obtained from Pierce Biotechnology. CyDye DIGE Fluors were obtained from GE Healthcare. Rabbit anti-PCNA polyclonal antibody was obtained from Beijing Protein Innovation, Ltd.; rabbit anti-nNOS and goat anti-biotin were obtained from Cell Signaling Technology. All other antibodies used in this study were purchased from Santa Cruz Biotechnology.

### Cell culture and treatments

Human neuroblastoma SH-SY5Y and HeLa cervix carcinoma cell lines were obtained from Type Culture Collection (China Center). SH-SY5Y and HeLa cells were cultured in DMEM/F12 and DMEM (Gibco), respectively, and supplemented with 10% (v/v) fetal bovine serum, 0.1 U/ml penicillin, and 0.1 mg/ml streptomycin (PAA) at 37°C in a humidified 5% CO_2_ atmosphere.

The SH-SY5Y cells were transferred to a low serum medium that contained 0.5% FBS for 12 h prior to further treatment. The rotenone solution was freshly prepared prior to each experiment in dimethyl sulfoxide (DMSO), and DMSO served as the vehicle control. L-NMMA, a NOS inhibitor, was dissolved in deionized water and added to the culture media at the final concentration of 0.5 mM 4 h prior to rotenone treatment. The final volume ratio of vehicle or drug solution to the media was controlled within 0.05%.

### Generation and detection of NO

S-Nitrosocysteine (SNOC) was used to generate NO according to previous methods [33] and was freshly prepared prior to each experiment. DAF-FM DA was used as a fluorescent indicator of intracellular NO to monitor the NO generated in response to cell treatment, according to the protocol provided by Molecular Probes. Following the cell treatment, the SH-SY5Y cells were washed with phosphate-buffered saline (PBS), followed by the addition of 5 μM DAF-FM DA for 20 min at 37°C. The cells were then rinsed and maintained in PBS, and the fluorescence within the cells was detected at λ_ex_ = 495 nm and λ_em_ = 515 nm using a Tecan Safire5 microplate reader.

### Western blot

The protein samples were dissolved in lysis buffer that contained 50 mM Tris-HCl, pH 7.4, 2% SDS, 10 mM dithiothreitol (DTT), and 5 mM EDTA with supplements of proteinase inhibitors. The denatured proteins were separated on a 12% SDS—PAGE, and the separated proteins were transferred onto a polyvinylidene difluoride membrane. After blocking and washing, the membrane was incubated with the indicated primary antibody followed by incubation with horseradish-linked secondary antibody. The immuno-recognition signals were detected by chemiluminescence using ImageQuant ECL (GE Healthcare), and the chemiluminescence was quantified using PDQuest software version 8.0.1.

### Annexin V-FITC/propidium iodide (PI) dual staining assay

SH-SY5Y cells treated with or without 500nM rotenone for 16h were trypsinized and the cell pellets were collected. The cells were resuspended in 100 μL binding buffer at 1 × 10^6^ cells/mL, and incubated with 5 μL annexin V-FITC and 10 μL PI for 15 min in the dark at room temperature. Then, these cells were added into 400 μL binding buffer and measured by FACSCalibur flow cytometer (Becton Dickinson).

### Biotin switch technique

The biotin switch technique (BST) was performed according to a previous report [34]. Cell lysates in the HENTS buffer that contained 250 mM Hepes, pH 7.4, 1 mM EDTA, 0.1 mM neocuproine, 0.1% SDS, and 1% Triton X-100 were incubated with 20 mM MMTS at 50°C for 30 min to block free thiol groups, and the excess MMTS was removed by acetone precipitation. The treated lysates were further incubated with 5 mM ascorbate and 1 mM biotin-HPDP for 1 h at room temperature. The biotinylated proteins were pulled down with NeutrAvidin agarose beads. The pellets were washed with neutralization buffer that contained 20 mM Hepes, pH 7.4, 100 mM NaCl, 1 mM EDTA, and 0.5% Triton X-100; the pellets were then eluted by Laemmli sample buffer followed by Western blot analysis.

### CyDye switch and 2D-DIGE

The combination of CyDye switch and DIGE was used to enrich and separate the SNO proteins in the SH-SY5Y cells. The cell lysates were incubated with 4X volume blocking buffer that contained 250mM HEPES-NaOH, pH7.4, 1mM EDTA, 0.1mM Neocuproine, 2.5% SDS, and 20mM MMTS, and the incubation was performed at 50°C for 30 min in the dark with vortex every 5 min. After the free thiols of the cell lysates were blocked and the S-nitrosothiols were reduced, the excess ascorbate was removed by acetone precipitation, and the precipitated proteins were dissolved in CyDye labeling buffer, which contained 30 mM Tris-HCl, 7 M urea, and 4% CHAPS, pH 8.0. CyDye DIGE Fluor dyes were used to label proteins at the final concentration of 10 μM. The labeling reaction was quenched by 2X CyDye labeling buffer that contained 2% v/v IPG buffer for pH 3–10 and 130 mM DTT. In the CyDye switch/2D-DIGE experiment, inverse strategy was adopted. On one 2DE running, the control sample labeled with Cy5 was the SH-SY5Y cell without rotenone treatment, while the internal standard sample labeled with Cy3 was a mixture, in which equal quantities of the protein lysates from the SH-SY5Y cells treated with and without rotenone were well mixed. The pooling of the equal quantities of the control sample and the internal standard sample was loaded to electrophoresis. Following 2DE, the gel was scanned by a Molecular Imager PharosFX System (Bio-Rad) with λ_ex_ = 548 nm and λ_em_ = 560 nm for the Cy3-labeled sample and λ_ex_ = 641 nm and λ_em_ = 660 nm for the Cy5-labeled sample, and the relative fluorescence intensity for a 2DE spot was estimated by the ratio of Cy5 against Cy3. Inversely, on the other 2DE running, the rotenone sample labeled with Cy5 was the SH-SY5Y cell treated with rotenone, while the internal standard sample labeled with Cy3 was a mixture of equal quantities of the control and rotenone sample. The sample loading and spot intensity estimation were the same as above. Since the composition in the internal standard sample was consistent, the fold-change for each spot could be obtained by comparison of the relative fluorescence intensity. The florescence images were analyzed using PDQuest software version 8.0.1. All the CyDye switch/2D-DIGE experiment, the related proteomic analysis and protein identification were conducted in three independent experiments.

### Tryptic digestion and protein identification by mass spectrometry

The selected 2D-DIGE spots were collected and subjected to in-gel digestion with trypsin following the protocol previously described [35]. The peptides generated by the tryptic digestion were identified by a micrOTOF-Q (Bruker Daltonics) mass spectrometer using the following instrument settings: nebulizer gas, nitrogen, 1.6 bar; dry gas, nitrogen, 6 l min^−1^, 190°C; capillary, −5,500 V (+4,000 V); end plate offset, −500 V; funnel 1 RF, 200 Vpp; funnel 2 RF, 200 Vpp; in-source CID energy, 0 V; hexapole RF, 100 Vpp; quadrupole ion energy, 5 eV; collision gas, argon; collision energy, 10 eV; collision RF 200/400 Vpp (timing 50/50); transfer time, 70 μs; prepulse storage, 5 μs; pulse frequency, 10 kHz; and spectra rate, 3 Hz. The MS/MS spectra data were searched against the SwissProt database using Data Analysis software (Bruker Daltonics) and the MASCOT in-house search engine (MatrixScience).

To identify the proteins in the PCNA interactome, immunoprecipitates from SH-SY5Y cells were subjected to gel-assisted digestion as previously described [36]. The tryptic peptides were delivered to a model LC–20AD NANO (Shimadzu) coupled with a micrOTOF-Q mass spectrometer equipped with a nanospray source. The peptides were eluted using a linear gradient of 5–80% acetonitrile with 0.1% formic acid. Each full MS scan was followed by three MS/MS scans of the three most intense ions, with data-dependent selection using the dynamic exclusion option. The mass spectrometer settings and spectra analysis method were the same as those used in the spot identification.

### Immunofluorescence staining

PCNA subcellular localization in the SH-SY5Y or HeLa cells was observed with a standard immunofluorescence staining protocol [37]. Cells on glass cover slips were fixed in PBS that contained 4% paraformaldehyde for 20 min on ice and permeabilized with 0.3% Triton X-100 for 5 min at room temperature, followed by incubation with primary antibodies overnight at 4°C. The cells were then treated with FITC or TRITC-labeled secondary antibodies and DAPI (for nuclear staining). The fluorescence images were viewed under an FV 500 confocal microscope (Olympus).

### Enrichment of nuclear and cytosolic fractions

The cell pellet was washed twice with ice-cold PBS and resuspended in 5X volume of ice-cold isolation buffer containing 20 mM Hepes—KOH, pH 7.5, 10 mM KCl, 1.5 mM MgCl_2_, 1 mM sodium EDTA, 1 mM sodium EGTA, 1 mM dithiothreitol, 0.1 mM PMSF, and protease inhibitors. The resuspended cells were broken by passing several times through a G26 needle. The homogenate was centrifuged at 1000 g for 15 min to precipitate the nuclear fraction, and the supernatant was further centrifuged at 10^5^ g for 30 min in L-100 XP ultracentrifuge (Beckman). The resulting supernatant was used as the cytosol fraction.

### Coimmunoprecipitation (CoIP) and pull-down assay

The SH-SY5Y cytosol was kept in isolation buffer for 30 min at 4°C. Following centrifugation at 12,000 g for 20 min, the supernatants were incubated with anti-PCNA antibody to immunoprecipitate PCNA-interacting proteins. The eluted immunoprecipitates were identified by LC-MS/MS or loaded onto 12% SDS-PAGE gels followed by Western blotting using anti-caspase-9 antibody as the primary antibody.

Using the pET system as indicated, wild-type or mutated recombinant His-PCNA proteins were expressed in *Escherichia coli* BL21 (DE3) cells. The His-tagged fusion proteins were purified with nickel-agarose beads (QIAGEN) according to the manufacturer’s instructions. Approximately 50 μg His-PCNA protein were incubated overnight at 4°C with the lysates obtained from HeLa. Following incubation, nickel-agarose beads were added to capture the recombinant protein and the proteins that bound to it. The PCNA-interacting proteins were identified by Western blot.

### Generation of wild-type and site-directed mutagenesis recombinant proteins

The wild-type PCNA sequence was amplified by PCR, using 5’-CGCGGATCCATGTTCGAGGCGCGCCTG-3’ (forward) and 5’-CCGCTCGAGCTAAGATCCTTCTTCATCCTCGATC-3’ (reverse) primers, from the cDNA of the human hepatocarcinoma cell line Huh7 and was inserted into the expression vector pET-28a. Mutant PCNA plasmids were constructed using the mutated PCR primer pairs for C81A, 5’-GTCCAAAATACTAAAAGCCGCCGGCAATGAAGATATC-3’ (forward) and 5’-GATATCTTCATTGCCGGCGGCTTTTAGTATTTTGGAC-3’ (reverse), and for C162A, 5’-GCTGTTGTAATTTCCGCAGCAAAAGACGGAGTG-3’ (forward) and 5’-CACTCCGTCTTTTGCTGCGGAAATTACAACAGC-3’ (reverse). The double-mutant C81A/C162A plasmid was also constructed with the same mutated PCR primers. The mutated PCNAs were inserted into pET-28a for protein expression.

### Caspase-9 activation assay

HeLa cytosol was incubated with cytochrome c (Calbiochem), 1 mM dATP and the indicated exogenous PCNA for 30 min at 37°C. The cleavage of caspase-9 was detected by Western blot using a polyclonal antibody against caspase-9 [38].

### Overexpression of PCNA in SH-SY5Y cells

The PCR product of PCNA gene with cloning sites of BamH1 and Xho1 was inserted into pcDNA3.1(+) plasmid (Invitrogen) for generation of transfection vector and the pcDNA3.1-PCNA was transfected to SH-SY5Y cells using Lipofectamine 2000 (Invitrogen) according to the manufacturer’s instructions.

### Statistical analysis

All experimental data were generated in triplicate (at minimum). The data are presented as the mean ± SEM, and significant differences among the test groups were determined by Student’s *t* test.

## Results

### Generation of NO stress in SH-SY5Y cells using rotenone

**Fig 1:**
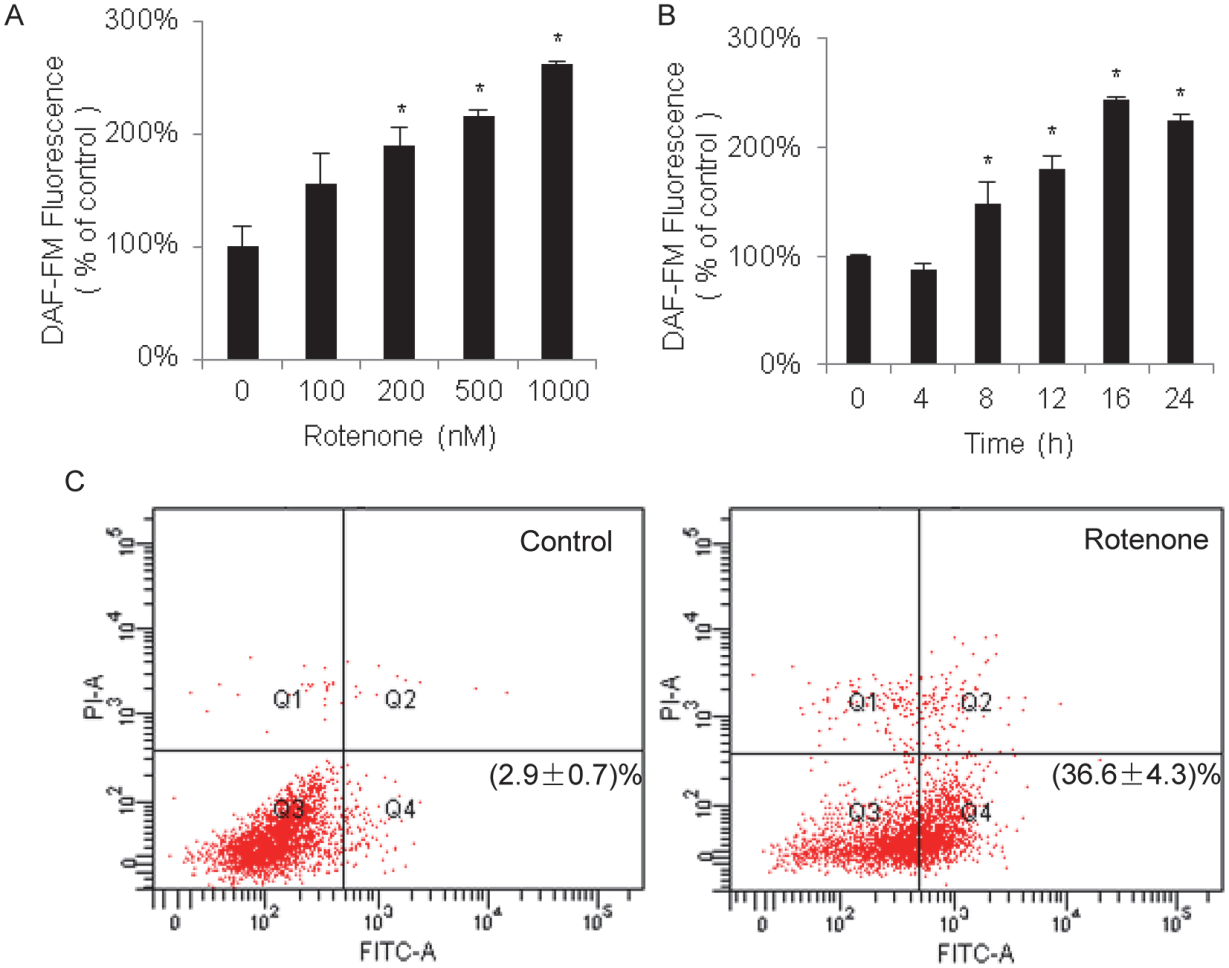
Phenotype changes in SH-SY5Y cells in response to rotenone treatment. The dose (A) and time (B) responses of NO generated in SH-SY5Y cells that were treated with rotenone were analyzed. 18h treatment (A) and 500nM rotenone (B) were used for the gradient experiments. The NO contents are represented as the ratio of the intensity of DAF-FM fluorescence in the rotenone-treated group compared with the vehicle-treated group (average ratio ± SEM, n = 3, *P<0.05). Apoptosis assessment by flow cytometry for SH-SY5Y cells treated with or without 500nM rotenone for 16h (C). Dot plot showed annexin V-FITC in x-axis and PI in y-axis. Cells in the fourth quadrant undergoing early stage apoptosis are annexin V-positive/PI negative. And cells at late stage apoptosis or necrotic cells are both annexin V-FITC and PI positive. The left represents the untreated cells as the control. The apoptotic rates are shown as the average ratio ± SEM (n = 3).

According to Fang et al., treatment with rotenone increases the NO content in SH-SY5Y cells, resulting in events that pathologically mimic the apoptosis of dopaminergic neurons in PD [19]. We followed their protocol with some modifications, especially regarding the incubation period and dosage. DAF-FM DA, a fluorescent probe actively conjugated with NO, was used to monitor the NO content within the cells. As depicted in Fig. 1A and 1B, the DAF-FM DA signals increased in a dose- and time-dependent manner after incubation with rotenone. The results revealed that the rotenone treatment to SH-SY5Y cells induced the NO accumulation within the cells. To ensure that the SH-SY5Y cells could tolerate NO stress and still maintain cell viability, we established a standard protocol for the cell treatment with rotenone through whole study, in which the SH-SY5Y cells were treated with 500 nM rotenone for 16 h. We further examined whether the increased NO content resulted from the abundance changes in nNOS. As shown in S1 Fig., treatment of cells with rotenone had no significant effect on the nNOS abundance, which implies that changes in the NO content of the SH-SY5Y cells were not directly related to the nNOS expression status. Several groups have observed apoptotic responses to rotenone treatment in SH-SY5Y cells. We explored the apoptotic rate of SH-SY5Y cells treated with 500nM rotenone for 16 h using annexin V-FITC and PI dual staining, which is an approach to monitor the membrane externalization of phospholipid phosphatidylserine occurs in the early stage of apoptosis. The data of flow cytometry shown in Fig. 1C exhibited that the stained cells after rotenone treatment significantly moved to the apoptotic phase (from Q3 to Q4) with the apoptosis rate at 36.6 ± 4.3% against control cells with the rate at 2.9 ± 0.7%. The evidence obtained from the typical apoptosis indicator revealed that rotenone caused the SH-SY5Y cells to undergo apoptosis. Taken together, these data indicate that rotenone treatment increased both NO stress and apoptosis in the SH-SY5Y cells.

### S-Nitrosoproteome in SH-SY5Y cells analyzed by CyDye switch/2D-DIGE

**Fig 2:**
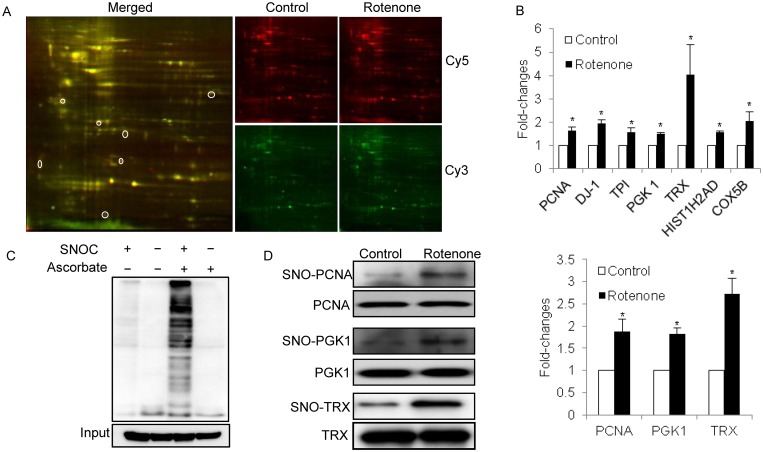
Analysis of S-nitrosoproteomes in SH-SY5Y cells with and without rotenone treatment using CyDye switch/2D-DIGE and BST/Western blot. (A) The representative 2D-DIGE images. The internal standard (Cy3, green) and the target samples (Cy5, red) have been defined in the Methods section. The gel images were acquired by fluorescence scanning at λ_ex_ = 548 nm and λ_em_ = 560 nm for Cy3-labelled samples and at λ_ex_ = 641 nm and λ_em_ = 660 nm for Cy5-labelled samples. In the merged image, the circled spots represent the spots that differentially responded to rotenone treatment. (B) Comparison of the spot volumes for the seven differential proteins on 2D-DIGE. The spot fold-changes corresponding to the differential spots on Fig. 2A were analyzed using PDQuest software version 8.0.1. The differential spots were defined as changes in spot volume over 1.5-fold in all the cases, and were excised and tryptic digested for protein identification with mass spectrometry (fold-changes of spot volumes ± SEM, n = 3, *P<0.05 versus vehicle-treated group). (C) Specificity and efficiency of biotin switch technique. SH-SY5Y cell lysates were treated with or without 200 μM SNOC followed by BST. Protein extract was loaded onto an SDS-PAGE. Western blot analysis was carried out, and the membrane was probed with anti-biotin. Control samples were subjected to PBS or SNOC but not to ascorbate. (D) Verification of the proteins with increased S-nitrosylation in SH-SY5Y cells treated with rotenone treatment. The proteins were extracted from SH-SY5Y cells with and without rotenone treatment and subjected to BST, and the biotinylated proteins were pulled down. The potentially S-nitrosylated proteins were examined by Western blot using the corresponding antibodies. The fold-changes are shown in the bar chart (n = 3, *P<0.05 versus vehicle-treated group).

CyDye switch/2D-DIGE is a common approach to analyze S-nitrosoproteomes [39–41]; in this approach, biotin tags are replaced by fluorescent tags to label SNO proteins. This method was recently employed to study protein S-nitrosylation in microglial cells [42]. To reduce the background noise, we optimized the approach by setting an internal standard as described in “Methods”. Approximately two hundred fluorescent spots were visualized on the images based on analysis using PDQuest software (Fig. 2A). Of the spots that had an intensity change over 1.5-fold due to rotenone treatment in three independent experiments, 7 spots showed an increased fluorescent signal, indicating these proteins had sensitivity to NO stress and were readily S-nitrosylated. The 7 spots were excised from the gels and transferred to the micrOTOF-Q mass spectrometer for protein identification following trypsin digestion. The differential S-nitrosylated proteins and their fold-changes are summarized in Fig. 2B, and all peptides matched with these proteins are listed in S1 Table.

Next, the BST was performed. Its specificity in SH-SY5Y cells was demonstrated (Fig. 2C). Western blot analysis of protein S-nitrosylation in SH-SY5Y showed a strong anti-biotin immunoreactivity for biotinylated proteins only in cells treated with SNOC, a NO-generating reagent, but not in untreated control cells, indicating a complete blocking of free cysteine thiols by MMTS and minimal endogenous S-nitrosylation in untreated cells. The absence of a signal in reaction without ascorbate demonstrates that the modification is SNO-specific. After establishing the efficacy and specificity of the BST in SH-SY5Y cells, three interesting proteins with increased S-nitrosylation, PCNA, PGK1, and TRX, were further examined by BST/Western blot. The lysates from the SH-SY5Y cells in the control or rotenone groups were subjected to BST followed by Western blot using the specific antibodies against the antigens. As shown in Fig. 2D, the three proteins were readily detectable by the corresponding antibodies in the lysates; however, no clearly different immuno-signals were observed between the control and rotenone groups. In the lysates that had been incubated with avidin, all immuno-signals in the cells treated with rotenone appeared remarkably augmented compared with the control cells. Thus, the Western blotting results were consistent with the CyDye switch/2D-DIGE results; some proteins in the SH-SY5Y cells were sensitive to NO stress and showed significantly increased S-nitrosylation.

### Localization of PCNA in SH-SY5Y cells

**Fig 3:**
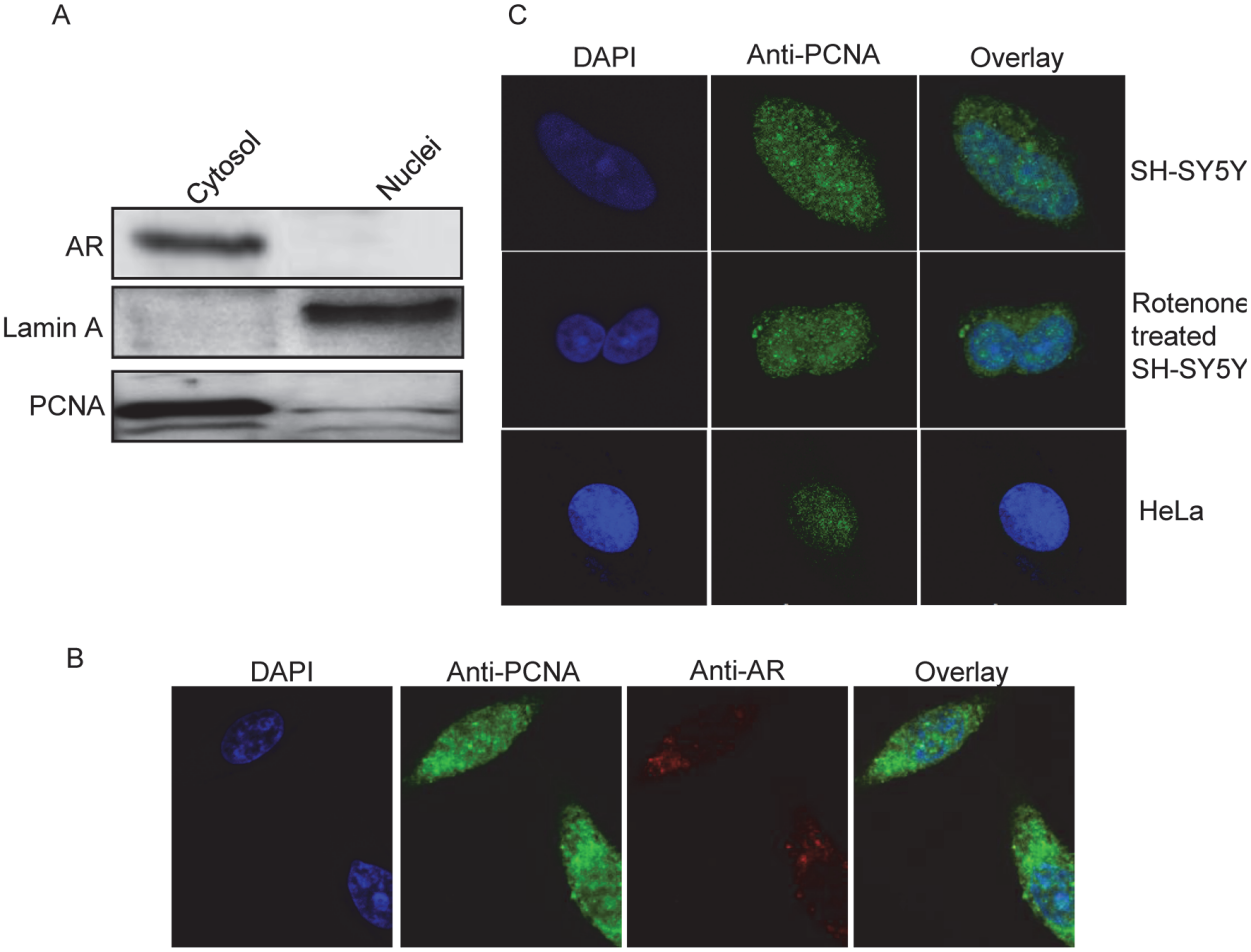
Localization of PCNA in SH-SY5Y cells. (A) Localization of PCNA in the cytosolic and nuclear fractions of SH-SY5Y cells was examined by Western blot with antibodies against PCNA, Lamin A (nuclei marker), and aldose reductase (AR, cytosolic marker). (B) Localization of PCNA in SH-SY5Y cells was observed by confocal microscopy. Signal for aldose reductase served as cytosolic marker. (C) Effect to the localization of PCNA in SH-SY5Y cells by rotenone treatment was monitored by confocal microscopy with immunofluorescence using the PCNA antibody. DAPI was used as a nuclei stain.

PCNA, a processivity factor for DNA polymerase ε in eukaryotic cells, is predominately located in the nucleus and involved in the apoptotic pathway. Recently, PCNA was also reported to be located in the cytosol of leukocytes [43]. We investigated the localization of PCNA in SH-SY5Y cells using Western blot and confocal microscopy. For the Western blot analysis, SH-SY5Y cells were fractioned into cytosolic and nuclear fractions; the presence of PCNA detected using an antibody specific for PCNA. The Western blot image in Fig. 3A indicates that PCNA in SH-SY5Y cells is dominantly located in the cytosol, not in the nucleus. In the confocal microscopy analysis (Fig. 3B), we stained PCNA and aldose reductase, a cytosolic marker, in SH-SY5Y cells, and observed that aldose reductase was apparently only cytosolic localization but PCNA was detectable in two localizations, cytosol and nuclei. The immuno-signals for PCNA and DAPI staining also do not overlap in SH-SY5Y cells, whereas they strongly overlap in HeLa cells, a positive control for the nuclear localization of PCNA (Fig. 3C). These findings indicate that PCNA in SH-SY5Y cells is primarily distributed in the cytosol and not in the nuclei. We further determined that the location of PCNA in SH-SY5Y cells was unaltered by rotenone treatment (Fig. 3C). Therefore, if PCNA involves apoptosis in SH-SY5Y cells, it is via the apoptosis pathways in the cytosol.

### S-nitrosylation regulation of the interaction of PCNA and caspase-9

**Fig 4:**
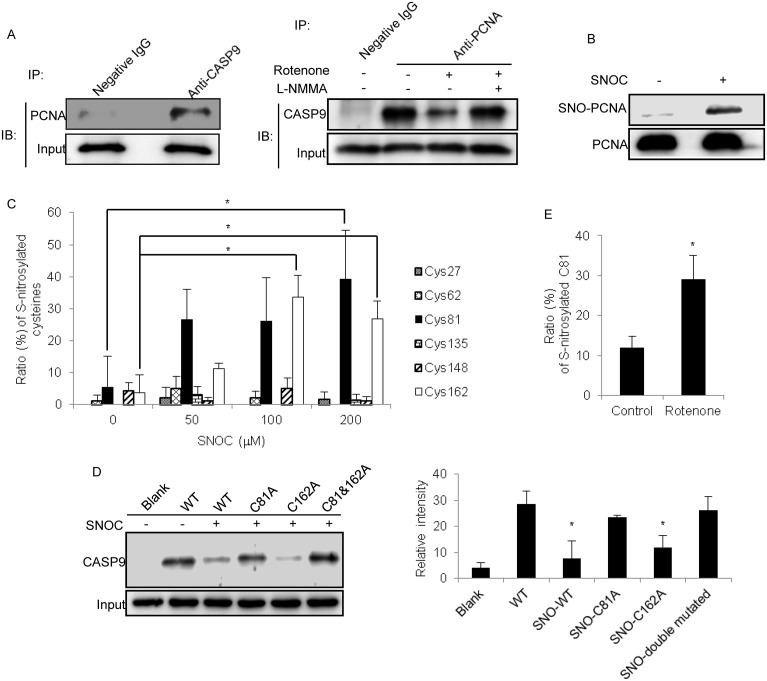
Effects of the S-nitrosylation status of PCNA on the interactions of PCNA and caspase-9. (A) Diagonal CoIP using two antibodies against PCNA and caspase-9 in SH-SY5Y cells. The interactions of PCNA and caspase-9 were negatively correlated with the NO contents in SH-SY5Y cells. L-NMMA was used for the inhibition of nNOS, and rabbit IgG was used as a negative control for immunoprecipitation. (B) The S-nitrosylation of recombinant PCNA, identified by BST/Western blot, using SNOC as a NO donor. (C) Comparison of the sensitivity of the potential cysteine residues of recombinant PCNA to S-nitrosylation under different NO stress levels. The sensitivity of cysteine residues to NO modification is represented as the ratios of the S-nitrosylated peptides identified by LC-MS/MS to the sum of the corresponding peptides, which include all S-nitrosylated and non-S-nitrosylated peptides at certain sites (n = 3, *P<0.05). (D) Effects of the PCNA mutants under NO stress on the interactions of PCNA and caspase-9. In the pull-down experiment, the wild-type PCNA and three PCNA mutants, PCNA-C81A,-C162A and-C81A/C162A, were treated with SNOC and incubated with the HeLa cytosol, followed by enrichment with nickel-agarose beads and detection with Western blot using an antibody against caspase-9. The left panel shows the Western blot image, and the right panel presents the interaction of caspase-9 with different SNOC-modified recombinant PCNAs. The relative immune-recognition intensities were estimated based on the ratios of the specific band volume against the total band volumes for caspase-9 in the upper panel (n = 3, *P<0.05 versus WT PCNA). (E) Comparison of the S-nitrosylated status of PCNA at Cys81 in SH-SY5Y cells with and without rotenone treatment. The S-nitrosylation status of PCNA at Cys81 is represented as the ratios of the S-nitrosylated Cys81 peptide to the sum of the peptides that contained Cys81, which were identified by LC-MS/MS (n = 3, *P<0.05).

How does cytosolic PCNA perform its biological functions in SH-SY5Y cells, especially under NO stress? To answer this question, we searched for proteins that interact with PCNA. CoIP with antibody against PCNA was conducted using the cytosolic fraction of SH-SY5Y cells, and the proteins that interacted with cytosolic PCNA were identified by LC-MS/MS. As shown in S2 Table, 76 proteins were identified as potential PCNA-interacting candidates, including heat shock protein 90, peroxiredoxin-6, and 14–3–3 protein. Caspase-9, a key caspase that initiates the apoptotic pathways in the cytoplasm, was of particular interest. We subsequently designed experiments to verify the proteomic observations. First, we conducted a diagonal CoIP using two antibodies against PCNA and caspase-9 in SH-SY5Y cells. As depicted in Fig. 4A, caspase-9 was found in the precipitate pulled down by the PCNA antibody. Similarly, PCNA was found in the precipitate pulled down by the caspase-9 antibody. These two CoIP results provided convincing evidence that the two proteins could physically interact with each other in the cytosol of SH-SY5Y cells. Second, we sought to determine whether the interaction between PCNA and caspase-9 could be affected by NO stress. As shown in the Western blot in Fig. 4A, the interaction between caspase-9 and PCNA was attenuated by treatment with rotenone. Moreover, the interaction between caspase-9 and PCNA was restored when the NO stress induced by rotenone was reduced by the addition of L-NMMA. Furthermore, the S-nitrosylation status of caspase-9 was carefully evaluated, and no change was observed under rotenone treatment of SH-SY5Y cells (S2 Fig.). Thus, the interaction between PCNA and caspase-9 is partially regulated by the S-nitrosylation of PCNA.

There are 6 cysteine residues in PCNA. Which cysteine residue(s) of PCNA is sensitive to NO stress? Considering that the amount of native PCNA immunoprecipitated from SH-SY5Y cells is rather limited, we generated a full-length recombinant PCNA to identify its S-nitrosylation site(s) in vitro. After the recombinant PCNA was incubated with SNOC, its S-nitrosylation status was examined using BST/Western blot. Fig. 4B shows that the recombinant PCNA is clearly S-nitrosylated after exposure to 200 μM SNOC for 30 min. For the sake of which cysteine residues are reactive to NO stress, the recombinant PCNA was treated with various concentrations of SNOC followed by BST labeling. The biotinylated PCNA was digested under non-reductive conditions, and the resulting peptides were subjected to LC-MS/MS. The status of the cysteine modification by MMTS (+46 atomic mass units, free sulfhydryl group) or biotin-HPDP (+428 atomic mass units, S-nitrosylated sulfhydryl group) could be clearly distinguished by MS/MS spectra. With selecting biotin-HPDP(C) and MMTS as the variable modifications in MASCOT, the search results could find the MS/MS spectra corresponding to the biotin-HPDP or MMTS labeled sites on peptides. On the basis of the search results, the spectra from labeled with biotin-HPDP and with MMTS were counted and the labeling efficiency for biotin-HPDP at a certain cysteine site was evaluated, termed as the S-nitrosylation ratio as illustrated in Fig. 4C. Fig. 4C showed all the cysteine residues on PCNA were possibly S-nitrosylated, however, the S-nitrosylation at the four residues, Cys27, Cys62, Cys135 and Cys148, appear the poor and inconsistent modification signal with the SNOC dose-independent mode. The two PCNA residues, Cys81 and Cys162, exhibited relatively higher S-nitrosylation ratios and a SNOC dose-dependent manner. Therefore, a deduction was logically elicited from the observation, which cysteine Cys81 and Cys162 are potentially reactive to NO. S3 Fig. showed the representative MS/MS spectra for the PCNA peptides that contained the biotinylated Cys81 and Cys162.

The results obtained via MS/MS were further verified by site-directed mutagenesis experiments. The Cys81 or Cys162 residue was substituted with alanine to yield recombinant PCNA mutants. The WT or mutant forms of PCNA were immobilized on nickel beads, exposed to SNOC or control conditions, and then incubated in the dark with a cytosolic fraction of HeLa cells, in which no PCNA was constitutively expressed. The amount of caspase-9 in the pull down was detected by Western blotting. As shown in Fig. 4D, wild-type PCNA treated with SNOC led to a decreased interaction between PCNA and caspase-9, while SNOC treatment of the C162A PCNA mutant resulted in the attenuation of the interaction of PCNA and caspase-9. This interaction was less affected by SNOC treatment of the C81A PCNA mutant. This finding implies that compared with Cys162, Cys81 of PCNA is more important for the PCNA interaction with caspase-9 and that the S-nitrosylation of PCNA can weaken this interaction.

We next considered whether the S-nitrosylation of Cys81 of PCNA occurs in SH-SY5Y cells. We employed BST in SH-SY5Y cells with and without rotenone treatment and labeled the SNO proteins with biotin-HPDP. The proteins were run on an SDS-PAGE gel, and the band that corresponded to the PCNA molecular mass was excised and in-gel digested by trypsin, followed by peptide identification with LC-MS/MS. The spectrum count was used to evaluate the protein abundance. Compared with SH-SY5Y cells without rotenone treatment, the percentage of the peptides that contained S-nitrosylated Cys81 was increased following rotenone administration (Fig. 4E). Thus, this PCNA cysteine residue in SH-SY5Y cells acts in a similar manner as in the in vitro response to NO stress.

### Regulation of caspase-9 activation by the S-nitrosylation of PCNA

**Fig 5:**
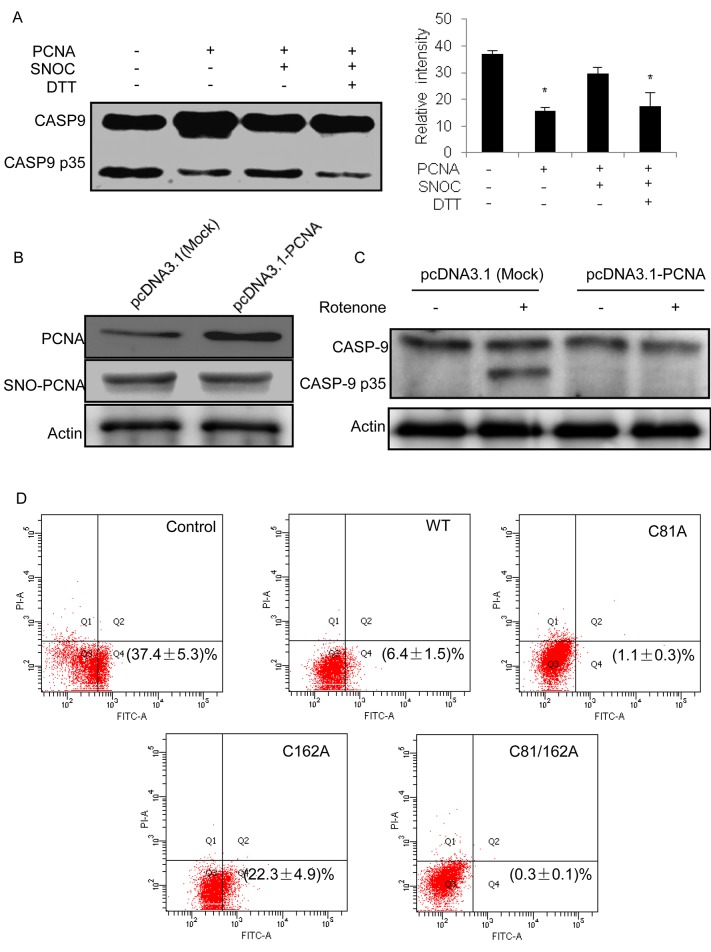
Regulation of caspase-9 activity by the S-nitrosylation status of PCNA. (A) Recombinant wild-type PCNA was immobilized onto nickel-agarose beads followed by incubation with and without SNOC. After the incubated beads were added to the HeLa cytosolic extracts, the activity of caspase-9 cleavage was measured by Western blot using an anti-caspase-9 antibody. As a control, DTT was used to reduce the S-nitrosylation of PCNA induced by SNOC. The relative intensities of cleaved caspase-9 are shown in the bar chart (n = 3, *P<0.05 versus blank group). (B) Western blot analysis to PCNA overexpression and S-nitrosylation status in SH-SY5Y cells that were transfected with pcDNA3.1-PCNA. (C) Caspase-9 cleavage assay in SH-SY5Y cells with or without PCNA overexpression. The cells were treated with 500 nM rotenone for 16h followed by the cleavage activity assay based upon Western blot using antibody against caspase-9. (D) Annexin V-FITC/PI dual staining assay for rotenone-induced apoptosis in the SH-SY5Y cells which were WT, C81A, C162A, and C81/162A PCNA overexpressed, respectively. The apoptotic rates are shown as the average ratio ± SEM (n = 3).

Caspase-9 activity can be regulated in many ways, such as phosphorylation by kinases or blockage by heat shock proteins. The activity of caspase-9 cleavage was evaluated using a HeLa cytosolic fraction, which contained apoptosome components, such as caspase-9 and APAF-1 [38]. In this in vitro assay, the addition of exogenous cytochrome c triggered APAF-1 oligomerization. Thus, caspase-9 was activated by its cleavage from procaspase to the active form. When purified recombinant PCNA was added to the reaction mixture, the amount of the cleavage product, 35 kD form, was remarkably reduced, which suggests that the interaction of PCNA with caspase-9 inhibited the caspase-9 activation (Fig. 5A). The inhibition of caspase-9 by PCNA was clearly prevented when SNO-PCNA was added to the reaction mixture. The inhibitory effect of PCNA was mostly restored when SNO-PCNA was reduced by DTT (Fig. 5A). The data obtained from the assay indicate that the interaction of PCNA and caspase-9 can partially block the activation of caspase-9, and S-nitrosylation of PCNA facilitates relieves this block. We next transfected pcDNA3.1-PCNA into SH-SY5Y cells and made the cells with overexpressed PCNA to track the corresponding changes of the caspase-9 cleavage activity. As shown in Fig. 5B, under the same treatment conditions of rotenone, S-nitrosylation status of PCNA in the cells with overexpressed PCNA was comparable with that in the cells with normal PCNA expression, whereas PCNA abundance in the two SH-SY5Y cells was significantly different. Furthermore, the inhibition extent to the caspase-9 cleavage activity in the SH-SY5Y cells with overexpressed PCNA was obviously enhanced compared with that in the cells with normal PCNA expression (Fig. 5C). This indicates that higher abundance of PCNA could partially eliminate the PCNA S-nitrosylation impact to the interaction of PCNA and casepase-9, resulting in inhibition to caspase-9 activation and reduction of apoptosis in the cells treated with rotenone. We further explored the rotenone-induced apoptosis rates of the SH-SY5Y cells, in which WT, C81A, C162A, and C81/162A PCNA were overexpressed, respectively. Compared with the apoptosis rate of control cells at 37.4 ± 5.3%, the data of flow cytometry shown in Fig. 5D exhibited that the SH-SY5Y cells with C81A and C81/162A overexpression were almost resistant to the rotenone-induced apoptosis with the rates at 1.1 ± 0.3% and 0.3 ± 0.1%, respectively, whereas the cells with WT and C162A overexpression showed the obviously different apoptosis rates, 6.4 ± 1.5% for WT and 22.3 ± 4.9% for C162A. As expected, in SH-SY5Y cell the overexpressed C81A or C81/162A PCNA that was insensitive to S-nitrosylation induced by NO under rotenone treatment could strengthen the interaction of PCNA and caspase-9 leading to apoptosis resistance. On the other hand, the apoptosis status in the cells with C162A overexpression was more serious than that in the cells with WT overexpression. Considering that PCNA C162A contained less one cysteine residue than WT, we deduced that the reducing status of cysteine residues in PCNA was another factor to contribute the caspase-9 related apoptosis. These results further supported our hypothesis that rotenone-induced apoptosis of SH-SY5Y cell was tightly correlated with the S-nitrosylation of PCNA-Cys81 in response to NO stress induced by rotenone, and offered other evidence that PCNA S-nitrosylation had a direct effect on the caspases-9-initiated apoptosis in the PD model.

## Discussion

It is generally accepted that a combination of genetic susceptibilities and environmental factors cause PD pathogenesis. In previous decades, several hypotheses for the selective neurodegeneration upon oxidative stress have been proposed to explain the relevant molecular mechanisms. ROS, the main free radicals, likely play an important role in PD, including the abnormalities of iron metabolism and protein modification under oxidative stress in the PD brain. NO is another important free radical species that were widely observed in PD animal models and PD patients. For instance, in Sprague-Dawley rats injected with rotenone, Yao et al. observed that nitrosative stress was augmented which led to increased parkin S-nitrosylation [44]; in mice injected with 6-OHDA or lipopolysaccharide (LPS), Singh et al. found that an augmentation of nitrite content was detected in both models, whereas the animal pretreated with a NOS inhibitor, N(G)-nitro-L-arginine methyl ester (L-NAME), exhibited protection against the lesions [45]; while in brain tissues of PD patients, Tsang et al. claimed that the S-nitrosylation of XIAP promoted apoptosis, and suggested nitrosative stress as a regulator to neuronal survival in PD pathogenesis [21]. As NO is an active free radical, it is generally accepted that NO could rapidly modify proteins and form nitrosylated or nitrated adducts. In PD animal models or PD patient brain tissues, several S-nitrosylated proteins were identified, such as PDI, Drp-1, Prx-2, XIAP, and parkin. The detailed mechanisms of NO stress and S-nitrosylated proteins to exert neurotoxicity in PD, nevertheless, remains poorly understood. Moreover, as one of the central molecules responsible for cell proliferation through regulation in DNA replication and repair, PCNA was found that its abnormal interaction with DNA polymerase β activity resulted in neuronal death in vivo, even though that the relevant mechanisms were not clarified yet [46]. What we addressed in the present study is to find the molecular basis that could partially explain the correlation between NO stress and PD neurodegeneration. Although in this study we conducted experiments in a PD cell model, the results gained under NO stress seem to delineate the potential mechanism of apoptosis in PD, which is pivotal in identifying the role of free radicals in PD as well as in designing therapeutic medicines for PD.

Herein, using the CyDye switch approach, we have successfully detected approximately 200 proteins that were likely S-nitrosylated in SH-SY5Y cells, including 7 proteins that showed increased S-nitrosylation in response to the increased NO stress induced by rotenone. Of these SNO proteins that are sensitive to NO stress, most have been observed as the S-nitrosylated adducts in previous studies. Protein DJ-1, a causative gene for familial PD, is a multi-functional protein involved in transcriptional regulation, molecular chaperoning and cell survival antagonization. Cys46 and Cys53 of DJ-1 are susceptible to S-nitrosylation; Cys46, which participates in the dimerization of DJ-1, is particularly susceptible [47]. Thioredoxin (TRX) is involved in various redox reactions and acts as an antioxidant through reversible oxidation. Cys69 and Cys73 of TRX can be S-nitrosylated in vitro [48]. Furthermore, SNO-TRX can positively modulate the apoptotic process, leading to an increased activity of apoptosis signal-regulating Kinase 1 (ASK1), a typical member of the mitogen-activated protein (MAP) kinase family [49]. Cytochrome c oxidase complex IV is a large transmembrane protein complex in mitochondria that receives electrons from cytochrome c molecules and transfers them to oxygen. Subunit 5B of this complex has been shown to be S-nitrosylated under excessive NO, which contributes to the apoptosis of pulmonary artery endothelial cells [50]. Triosephosphate isomerase (TPI) plays an important role in glycolysis by adjusting the rapid equilibrium between dihydroxyacetone phosphate and glyceraldehyde-3-phosphate. Phosphoglycerate kinase 1 (PGK1) is another glycolytic enzyme that catalyzes the reversible transfer of a phosphate group from 1, 3-bisphosphoglycerate to ADP and produces 3-phosphoglycerate and ATP. There is evidence indicating that the two glyco-metabolism enzymes are NO sensitive. SNO-TPI was identified in prostate epithelial cells [51], rat cardiac cells [52], and ischemia/reperfusion cardiac cells [53], whereas SNO-PGK1 was identified in myocardium [54]. However, the functions of the modified TPI and PGK1 remain unclear. S-nitrosylation of histone H2A has not been reported to date; in contrast, the S-nitrosylated form of PCNA was identified in prostate epithelial cells in vitro by Lam et al., although no functional analysis was performed in the proteomic screening study.

**Fig 6:**
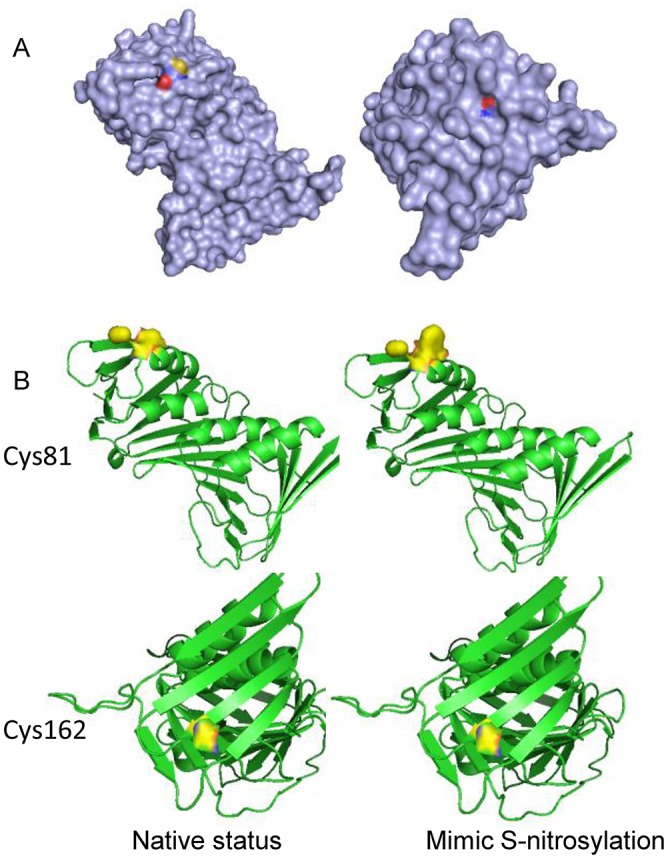
Potential structural consequences of S-nitrosylation for PCNA. (A) The surface accessibility of S-nitrosylation sites. The sulfhydryl group (yellow) of Cys81 was exposed at the protein surface (left), but that of Cys162 was buried inside (right). (B) Molecular simulations of wild-type and SNOC-modified PCNAs. S-nitrosylation at Cys81 clearly affected the local structure of this residue, which may interfere with the interactions, but the Cys162 modification had no significant influence on the structure.

PCNA was recently identified in the cytosol of several leukocytes and was determined to be an apoptotic regulator through its interaction with caspases [43]. The manner in which cytosolic PCNA participates in the regulation of apoptosis has not been clearly elucidated. PCNA may be regulated by posttranslational modifications. For example, Tyr211 of PCNA was identified as a phosphorylated residue. The phosphorylation at Tyr211 is required to maintain the protein function on chromatin and is dependent on the tyrosine kinase activity of the EGF receptor in the nucleus. The increased Tyr211 phosphorylation of PCNA induced by EGFR coincides with pronounced cell proliferation and is closely correlated with the poor survival of breast cancer patients [55]. PCNA can also be acetylated, which affects the subcellular localization and function of the protein. The acetylation of PCNA at Lys14 induced by UV irradiation results in the dissociation of PCNA and MutT homolog2 (MTH2) [56]. In general, the interaction between MTH2 and PCNA in the nucleus increases PCNA stability and facilitates DNA replication and repair. Once cells are exposed to UV light, the interaction between MTH2 and PCNA is disrupted, and PCNA degradation is accelerated. Under exogenous NO stress, SNO-PCNA was first detected in the lysate of prostate epithelial cells [51]. The authors attempted determine the biological significance of the modification with regard to the nuclear localization of PCNA, as well as the regulation of DNA replication. For the first time, we demonstrated that PCNA was specifically localized in the cytosolic fraction of SH-SY5Y cells. More importantly, the experimental evidence demonstrated that cytosolic PCNA was sensitive to NO stress in SH-SY5Y cells treated with rotenone. With the evidence from in vitro and the cell model experiments, we further confirmed the interaction of PCNA and caspase-9 as a critical step in the regulation of the apoptotic pathway, and provided a potential theoretical model to describe why the rotenone treatment led to apoptosis augmentation in SH-SY5Y cells. Both S-nitrosylation sites in PCNA are evolutionarily conserved across a wide variety of organisms. To investigate the potential structural consequences of S-nitrosylation, we performed molecular simulations of wild-type and SNOC-modified PCNA in silico using atomic co-ordinates from the 3TBL (PDB code) crystal structure [57]. We observed that the sulfhydryl group (with yellow) of Cys81 was exposed at the protein surface in unbound form (Fig. 6A, left), whereas the sulfhydryl group of Cys162 was buried inside (Fig. 6A, right). Considering the sensitivity of these cysteine residues to NO, the spatial accessibility on the molecular surface may facilitate site-specific S-nitrosylation, which is consistent with the results of Doulias et al. [58]. Cysteine-tryptophan substitution has been reported to be a suitable mutant to mimic S-nitrosylation [59]. Molecular simulations of the C81W mutant revealed significant conformational alterations in the spatial structure of PCNA, whereas no apparent change was observed in C162W (Fig. 6B), which suggests that an inhibition effect for the interactions with caspase-9 by PCNA S-nitrosylation may be introduced by the conformational changes caused by S-nitrosylated Cys81. The discovery of PCNA S-nitrosylation in this study offers two interesting findings to investigate the biological functions of PCNA. First, PCNA possesses cysteine residues that are sensitive to NO stress, and the modification status likely results in functional changes. This finding indicates that the oxidative stress induced by drug treatment can cause PCNA signaling cascades. Second, of the several proteins that interacted with PCNA and have been associated with functional regulation in the nucleus, the interaction of PCNA and caspase-9 may be useful for determining the regulation mode of apoptosis. In addition, the function of PCNA complexes in the cytosol may differ from those in the nucleus.

The activation of caspase-9 is an initial event in the intrinsic apoptosis pathway. It is generally accepted that the activation requires binding of its precursor, procaspase-9, to a complex of two proteins, APAF-1 and cytochrome c, and the self-cleavage specific aspartic residues of procaspase-9. Cleaved caspase-9 further processes other caspase members, including caspase-3 and caspase-7, which leads to the initiation of the apoptotic pathway. However, the activation of caspase-9 is restricted within the triple protein complex and mediated by several protein components or posttranslational modifications, such as HBXIP [60], TUCAN [61], ERK2 [62], and PKA [63]. XIAP has been reported to directly interact with caspase-9, and the binding of XIAP/caspase-9 has opposing effects on caspase activity and apoptosis [64]. Furthermore, previous studies have found that c-Abl binds directly to caspase-9 and the bound c-Abl phosphorylates caspase-9 on Tyr153 and further regulates the caspase-9-related apoptotic response to genotoxic stress [65]. With the exception that they are all kinases, these caspase-9-interacting proteins do not appear to have a specific biochemical function, as TUCAN is located on the ER and HBXIP and XIAP are related to virus proteins. This study contributed new proteins to the list of proteins that interact with caspase-9. Compared with the early observations, the interaction of PCNA and caspase-9 provides two important findings with regards to apoptosis. First, the apoptosome complex contains three key proteins, but it also has multiple associated proteins that interact with the apoptosome proteins based on cell types or biological stimuli. PCNA was shown to interact with caspase-9 in SH-SY5Y cells; however, whether the interaction exists globally in many cells must be addressed in future research. Because our data suggest that SNO proteins could exert influences on the apoptotic pathway, NO modification of the apoptosome should be considered in future research.

## Supporting Information

S1 Fig Detection of nNOS expression under the indicated treatments. SH-SY5Y cells in the low-serum media were pretreated with L-NMMA followed by the addition of rotenone. The levels of nNOS under the different treatments were detected by Western blot.
(TIF)

S2 Fig Evaluation of the potential S-nitrosylation for caspase-9 in SH-SY5Y cells treated with rotenone. The lysate of SH-SY5Y cells with and without rotenone treatment was obtained and subjected to BST. The biotinylated proteins were pulled down with NeutrAvidin beads and detected by Western blot using anti-caspase-9.
(TIF)

S3 Fig Representative MS/MS spectra for the PCNA peptides that contained the biotinylated Cys81 (upper) and Cys162 (lower). δC represents biotin-HPDP derivatized cysteine (+428), which was included in the b- or y-ion series.
(TIF)

S1 Table The up-regulated SNO-proteins of SH-SY5Y cells in response to rotenone treatment. (DOCX)

S2 Table The proteins potentially interact with cytosolic PCNA in SH-SY5Y cells. (DOCX)
