# Supplementary figures and images for: The S-Nitrosylation Status of PCNA Localized in Cytosol Impacts the Apoptotic Pathway in a Parkinson’s Disease Paradigm
Source: PLoS One. 2015 Feb 12;10(2):e0117546. doi: 10.1371/journal.pone.0117546 (PMC4326459; doi:10.1371/journal.pone.0117546)

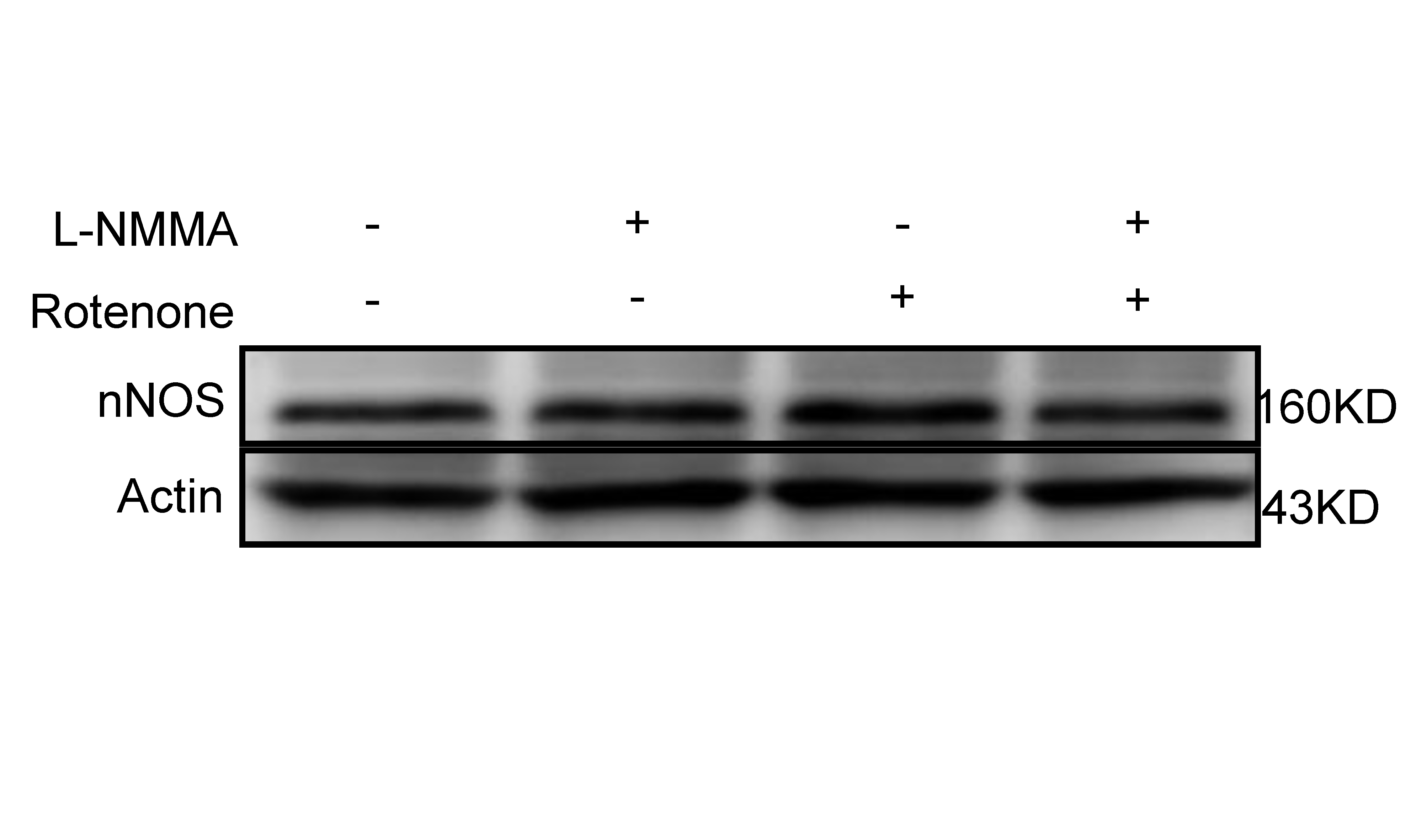

Supplement: S1 Fig — SH-SY5Y cells in the low-serum media were pretreated with L-NMMA followed by the addition of rotenone. The levels of nNOS under the different treatments were detected by Western blot. (TIF) [file pone.0117546.s001.tif]

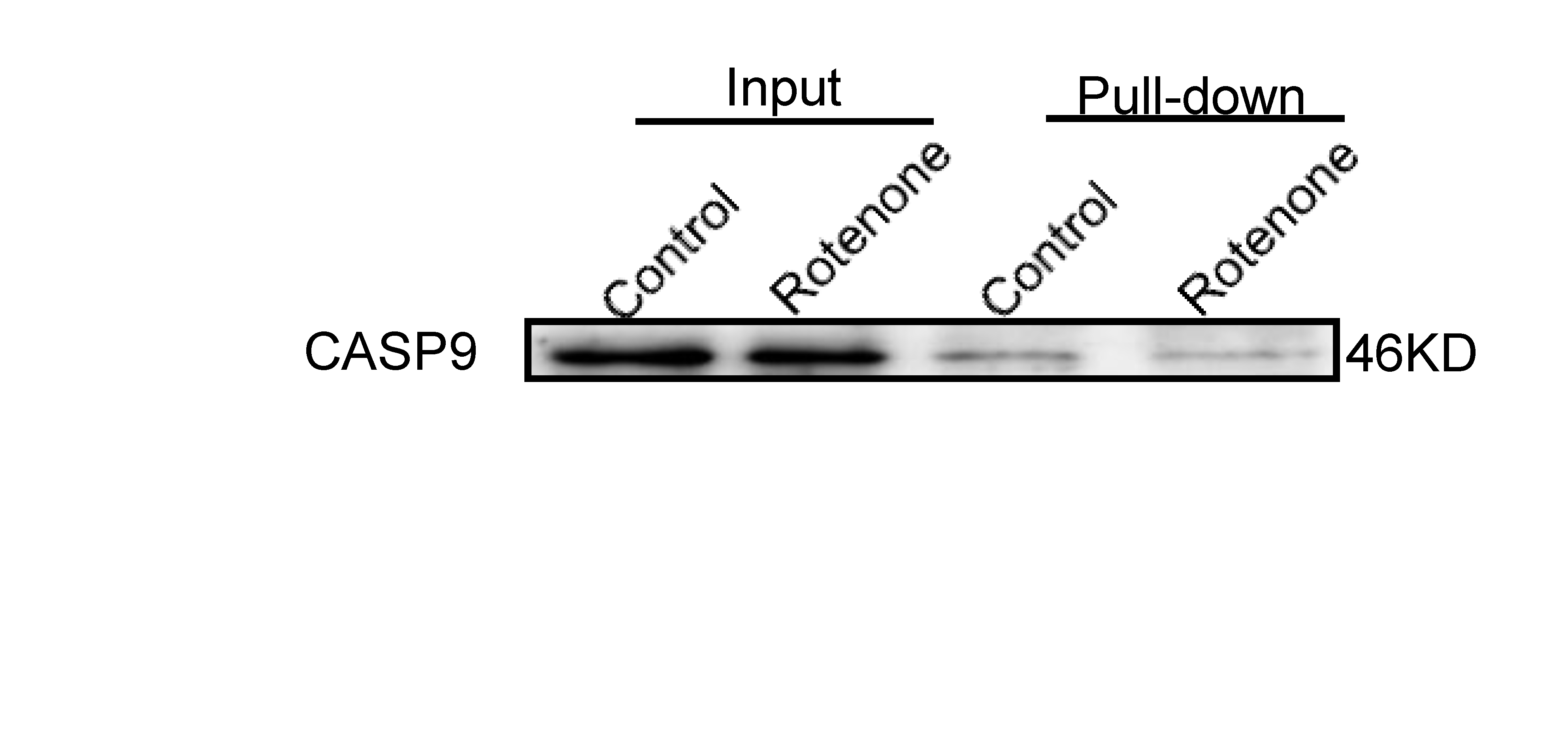

Supplement: S2 Fig — The lysate of SH-SY5Y cells with and without rotenone treatment was obtained and subjected to BST. The biotinylated proteins were pulled down with NeutrAvidin beads and detected by Western blot using anti-caspase-9. (TIF) [file pone.0117546.s002.tif]

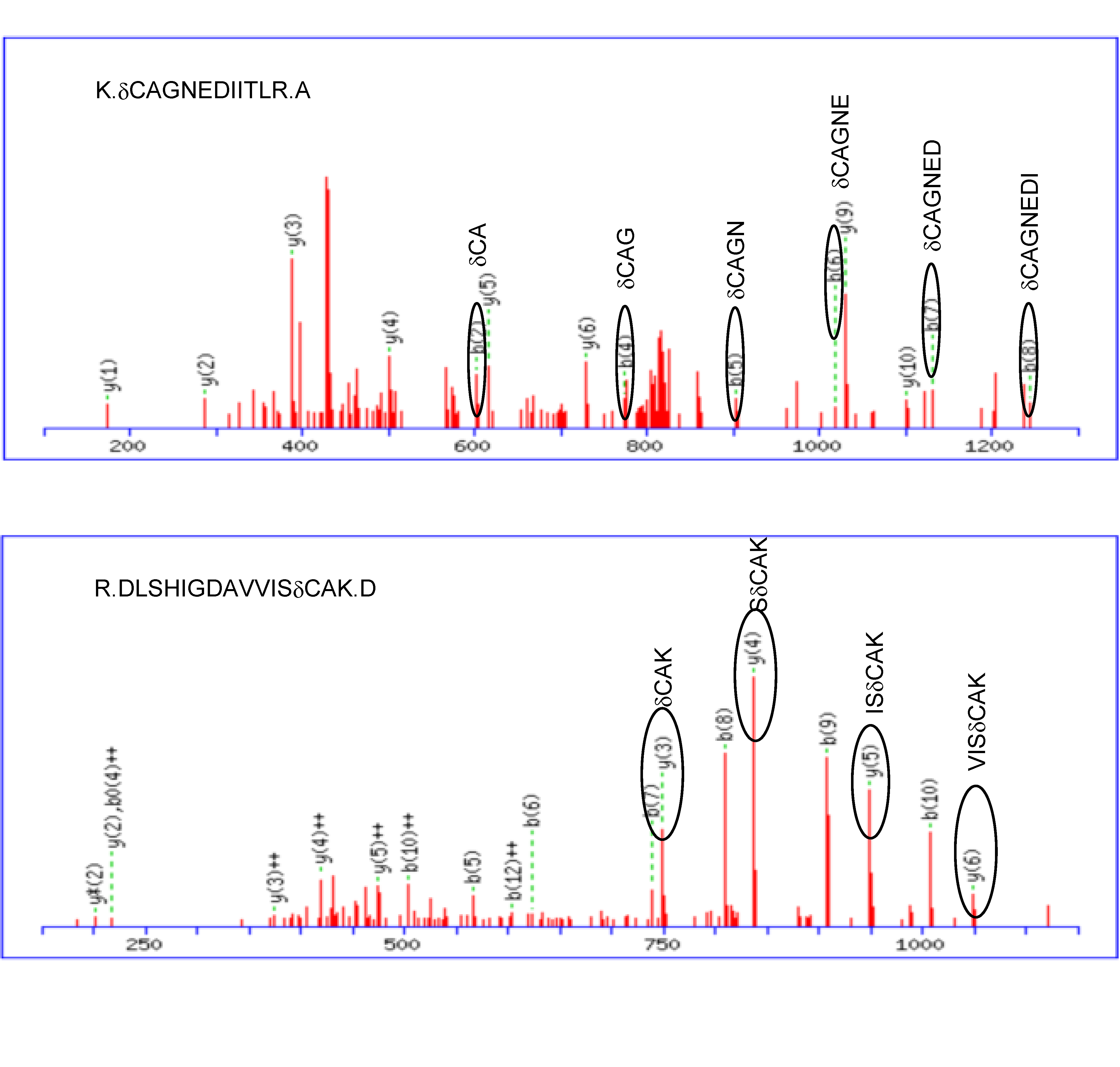

Supplement: S3 Fig — δC represents biotin-HPDP derivatized cysteine (+428), which was included in the b- or y-ion series. (TIF) [file pone.0117546.s003.tif]
