# Supplementary material for: The S-Nitrosylation Status of PCNA Localized in Cytosol Impacts the Apoptotic Pathway in a Parkinson’s Disease Paradigm
Source: PLoS One. 2015 Feb 12;10(2):e0117546. doi: 10.1371/journal.pone.0117546 (PMC4326459; doi:10.1371/journal.pone.0117546)
Supplement: S1 Table — (DOCX) [file pone.0117546.s004.docx]

**Supplementary Table 1:** The up-regulated SNO-proteins of SH-SY5Y cells in response to rotenone treatment

| Accession No. | Protein Name | No. peptides  matched | | Protein  score | Matched Peptides |
| --- | --- | --- | --- | --- | --- |
| P12004 | Proliferating cell nuclear antigen | | 20 | 1715 | K.YYLAPK.I |
|  |  | |  |  | R.LVQGSILK.K |
|  |  | |  |  | K.VSDYEMK.L |
|  |  | |  |  | K.IADMGHLK.Y |
|  |  | |  |  | K.MPSGEFAR.I |
|  |  | |  |  | R.YLNFFTK.A |
|  |  | |  |  | R.SEGFDTYR.C |
|  |  | |  |  | R.LVQGSILKK.V |
|  |  | |  |  | K.CAGNEDIITLR.A |
|  |  | |  |  | K.FSASGELGNGNIK.L |
|  |  | |  |  | R.NLAMGVNLTSMSK.I |
|  |  | |  |  | R.SEGFDTYRCDR.N |
|  |  | |  |  | K.YYLAPKIEDEEGS.- |
|  |  | |  |  | R.DLSHIGDAVVISCAK.D |
|  |  | |  |  | R.CDRNLAMGVNLTSMSK.I |
|  |  | |  |  | R.DLSHIGDAVVISCAKDGVK.F |
|  |  | |  |  | R.ICRDLSHIGDAVVISCAK.D |
|  |  | |  |  | R.AEDNADTLALVFEAPNQEK.V |
|  |  | |  |  | K.ATPLSSTVTLSMSADVPLVVEYK.I |
|  |  | |  |  | K.LMDLDVEQLGIPEQEYSCVVK.M |
| Q99497 | Protein DJ-1 | | 4 | 441 | K.APLVLKD.- |
|  |  | |  |  | K.VTVAGLAGKDPVQCSR.D |
|  |  | |  |  | R.DVVICPDASLEDAKK.E |
|  |  | |  |  | K.GAEEMETVIPVDVMRR.A |
| P60174 | Triosephosphate isomerase | | 14 | 2030 | K.FFVGGNWK.M |
|  |  | |  |  | K.IAVAAQNCYK.V |
|  |  | |  |  | K.SNVSDAVAQSTR.I |
|  |  | |  |  | K.LDEREAGITEK.V |
|  |  | |  |  | K.VIADNVKDWSK.V |
|  |  | |  |  | R.IIYGGSVTGATCK.E |
|  |  | |  |  | K.QSLGELIGTLNAAK.V |
|  |  | |  |  | R.HVFGESDELIGQK.V |
|  |  | |  |  | K.TATPQQAQEVHEK.L |
|  |  | |  |  | R.KQSLGELIGTLNAAK.V |
|  |  | |  |  | K.VVLAYEPVWAIGTGK.T |
|  |  | |  |  | K.VTNGAFTGEISPGMIK.D |
|  |  | |  |  | K.DCGATWVVLGHSERR.H |
|  |  | |  |  | K.VPADTEVVCAPPTAYIDFAR.Q |
| P00558 | Phosphoglycerate kinase 1 | | 14 | 1651 | K.AAVPSIK.F |
|  |  | |  |  | K.ELNYFAK.A |
|  |  | |  |  | K.ALMDEVVK.A |
|  |  | |  |  | K.DCVGPEVEK.A |
|  |  | |  |  | K.AEPAKIEAFR.A |
|  |  | |  |  | R.GTKALMDEVVK.A |
|  |  | |  |  | K.SLLGKDVLFLK.D |
|  |  | |  |  | K.ALMDEVVKATSR.G |
|  |  | |  |  | R.AHSSMVGVNLPQK.A |
|  |  | |  |  | K.LGDVYVNDAFGTAHR.A |
|  |  | |  |  | K.ACANPAAGSVILLENLR.F |
|  |  | |  |  | K.ALESPERPFLAILGGAK.V |
|  |  | |  |  | K.ITLPVDFVTADKFDENAK.T |
|  |  | |  |  | R.VDFNVPMKNNQITNNQR.I |
| P10599 | Thioredoxin | | 3 | 279 | K.LEATINELV.- |
|  |  | |  |  | K.TAFQEALDAAGDK.L |
|  |  | |  |  | K.MIKPFFHSLSEK.Y |
| P20671 | Histone H2A type 1-D | | 5 | 361 | R.HLQLAIR.N |
|  |  | |  |  | R.AGLQFPVGR.V |
|  |  | |  |  | R.NDEELNKLLGK.V |
|  |  | |  |  | R.HLQLAIRNDEELNK.L |
|  |  | |  |  | K.VTIAQGGVLPNIQAVLLPK.K |
| P10606 | Cytochrome c oxidase subunit 5B | | 2 | 106 | K.LVPQQLAH.- |
|  |  | |  |  | K.GLDPYNVLAPK.G |
