# Supplementary material for: The S-Nitrosylation Status of PCNA Localized in Cytosol Impacts the Apoptotic Pathway in a Parkinson’s Disease Paradigm
Source: PLoS One. 2015 Feb 12;10(2):e0117546. doi: 10.1371/journal.pone.0117546 (PMC4326459; doi:10.1371/journal.pone.0117546)
Supplement: S2 Table — (DOCX) [file pone.0117546.s005.docx]

**Supplementary Table 2:** The proteins potentially interact with cytosolic PCNA in SH-SY5Y cells

| Accession No. | | Protein Name | Accession No. | | Protein Name |
| --- | --- | --- | --- | --- | --- |
| P68104 | Elongation factor 1-alpha 1 | | Q16531 | DNA damage-binding protein 1 | |
| Q92616 | Translational activator GCN1 | | Q5T4S7 | E3 ubiquitin-protein ligase UBR4 | |
| P23921 | Ribonucleoside-diphosphate reductase large subunit | | Q7L2H7 | Eukaryotic translation initiation factor 3 subunit M | |
| P21333 | Filamin-A | | Q96T76 | MMS19 nucleotide excision repair protein homolog | |
| P14618 | Pyruvate kinase isozymes M1/M2 | | Q7L576 | Cytoplasmic FMR1-interacting protein 1 | |
| P08238 | Heat shock protein HSP 90-beta | | Q9UPQ0 | LIM and calponin homology domains-containing protein 1 | |
| P61978 | Heterogeneous nuclear ribonucleoprotein K | | P38919 | Eukaryotic initiation factor 4A-III | |
| Q14204 | Cytoplasmic dynein 1 heavy chain 1 | | P33993 | DNA replication licensing factor MCM7 | |
| P78371 | T-complex protein 1 subunit beta | | Q9BZZ5 | Cell migration-inducing gene 8 protein | |
| P13639 | Elongation factor 2 | | Q9UBT2 | Ubiquitin-like 1-activating enzyme E1B | |
| Q05639 | Elongation factor 1-alpha 2 | | P49368 | T-complex protein 1 subunit gamma | |
| O14980 | Exportin-1 | | P22234 | Multifunctional protein ADE2 | |
| P23528 | Cofilin-1 | | P47756 | F-actin-capping protein subunit beta | |
| O00429 | Dynamin-1-like protein | | P05198 | Eukaryotic translation initiation factor 2 subunit 1 | |
| P11586 | C-1-tetrahydrofolate synthase, cytoplasmic | | P27348 | 14-3-3 protein theta | |
| P60709 | Actin, cytoplasmic 1 | | P06493 | Cell division control protein 2 homolog | |
| P30041 | Peroxiredoxin-6 | | P28340 | DNA polymerase delta catalytic subunit | |
| P10599 | Thioredoxin | | P60228 | Eukaryotic translation initiation factor 3 subunit E | |
| Q15181 | Inorganic pyrophosphatase | | P68036 | Ubiquitin-conjugating enzyme E2 L3 | |
| P43246 | DNA mismatch repair protein Msh2 | | Q5VYK3 | Proteasome-associated protein ECM29 homolog | |
| P30153 | Serine/threonine-protein phosphatase 2A 65 kDa regulatory subunit A alpha isoform | | P62258 | 14-3-3 protein epsilon | |
| Q04637 | Eukaryotic translation initiation factor 4 gamma 1 | | Q9BRX2 | Protein pelota homolog | |
| P46783 | 40S ribosomal protein S10 | | P35080 | Profilin-2 | |
| P11142 | Heat shock cognate 71 kDa protein | | O00151 | PDZ and LIM domain protein 1 | |
| Q99832 | T-complex protein 1 subunit eta | | Q8NBS9 | Thioredoxin domain-containing protein 5 | |
| P63244 | Guanine nucleotide-binding protein subunit beta-2-like 1 | | P61764 | Syntaxin-binding protein 1 | |
| Q8WUM4 | ALG-2-interacting protein X | | Q92878 | DNA repair protein RAD50 | |
| P55211 | Caspase-9 | | Q15004 | PCNA-associated factor | |
| P16152 | Carbonyl reductase [NADPH] 1 | | Q9GZT9 | Egl nine homolog 1 | |
| P35221 | Catenin alpha-1 | | P37840 | Alpha-synuclein | |
| O43813 | LanC-like protein 1 | | Q9BUH6 | Uncharacterized protein C9orf142 | |
| P26641 | Elongation factor 1-gamma | | Q13813 | Spectrin alpha chain, brain | |
| Q9UL46 | Proteasome activator complex subunit 2 | | P61981 | 14-3-3 protein gamma | |
| Q13057 | Bifunctional coenzyme A synthase | | O94921 | Cyclin-dependent kinase 14 | |
| Q14152 | Eukaryotic translation initiation factor 3 subunit A | | Q9H3M7 | Thioredoxin-interacting protein | |
| P61289 | Proteasome activator complex subunit 3 | | Q9NS86 | LanC-like protein 2 | |
| P27816 | Microtubule-associated protein 4 | | Q9H254 | Spectrin beta chain, non-erythrocytic 4 | |
| P52907 | F-actin-capping protein subunit alpha-1 | | Q9UQ80 | Proliferation-associated protein 2G4 | |
